# RTP4 Suppresses Colorectal Cancer Progression via MHC‐I‐Mediated CD8^+^ T Cell Infiltration and Enhances Immunotherapy Response

**DOI:** 10.1111/jcmm.70915

**Published:** 2025-10-22

**Authors:** Chengpeng Yu, Yifan Li, Zhenzhe Liu, Wen Ye, Lin Wei, Weiqi Xu, Likun Yan, Haifeng Ye

**Affiliations:** ^1^ Department of General Surgery, The First Affiliated Hospital, Jiangxi Medical College Nanchang University Nanchang China; ^2^ Clinical College Nanchang Medical College Nanchang China; ^3^ Huan ‐ Kui Academy, Jiangxi Medical College Nanchang University Nanchang China; ^4^ The Second Clinical Medical College, Jiangxi Medical College Nanchang University Nanchang China; ^5^ Medical Center of Burn Plastic and Wound Repair, the First Affiliated Hospital, School of Basic Medical Sciences, Institute of Biomedical Innovation, Jiangxi Medical College Nanchang University Nanchang China

**Keywords:** biomarker, colorectal carcinoma, immunotherapy, RTP4, tumour microenvironment

## Abstract

While RTP4 is known to regulate odorant receptor trafficking, its role in colorectal cancer (CRC) remains unclear. This study investigates the clinical relevance and functional mechanisms of RTP4 in CRC. Comprehensive analyses revealed significant downregulation of *RTP4* expression in CRC tissues, correlating with poor patient prognosis. *RTP4* expression showed strong associations with immune‐related genes, biological processes and anti‐tumour immune cell infiltration. Mechanistic studies demonstrated that RTP4 upregulates MHC‐I expression, which enhances CD8^+^ T cell recruitment and strengthens anti‐tumour immunity in both cellular and animal models. Furthermore, RTP4 overexpression markedly improved the efficacy of immune checkpoint blockade therapy. All in all, our findings establish RTP4 as a dual‐functional biomarker for prognosis prediction and a potential target to enhance immunotherapy responsiveness in CRC.

## 1 Introduction

Colorectal cancer (CRC) is the third most prevalent and the second most lethal cancer worldwide [1, 2]. Although the 5‐year overall survival rate of CRC patients with stages I–III ranges from 68% to 90%, the rate in stage IV metastatic CRC (mCRC) patients is still low, which ranges from 11% to 14% [3]. According to the clinical characteristics and tumour molecular profiles, the first‐line treatment of mCRC relies on a combination of chemotherapy and targeted therapies [4]. Recently, immune checkpoint blockade (ICB) treatment has shown beneficial effects on CRC patients. However, only about 15% of CRC patients currently benefit from ICB treatment [5]. Several studies have found that CRC patients with deficient mismatch repair (dMMR) or microsatellite instability‐high (MSI‐H) are responsive to ICB treatment, while the majority of CRC patients with proficient mismatch repair (pMMR) or microsatellite stable (MSS) are unresponsive to ICB treatment [6, 7, 8]. One reason for this phenomenon is the inactivation and exhaustion of infiltrating CD8^+^T cells, leading to tumour immune evasion [9]. However, the exact molecular mechanisms of immune evasion in CRC are still unclear, and more therapeutic targets need to be discovered.

The RTP (receptor‐transporting protein) family consists of 4 members (RTP1‐4), and they were first found to promote cell surface expression of odorant receptors [10]. The general function of RTP family members is to promote cell surface expression of G‐protein–coupled receptors (GPCRs), such as odorant receptors that can mediate smell sensing [10]. As a member of the RTP family, RTP4 is expressed in several human tissues [11]. RTP4 was found to form a complex with the μ and δ opioid receptors and enhance their cell surface expression and function [12]. In addition, RTP4 may affect virus replication, and its expression can be induced upon viral infection [13, 14].

In recent years, several studies have reported that RTP4 may also play a role in cancer. In prostate cancer, *RTP4* methylation serves as a novel biomarker for clinical diagnosis and treatment [15]. In melanoma, the expression of *RTP4* was found to correlate with immune infiltrates and the expression of immune checkpoint encoding genes [16]. RTP4 was also implicated in pyroptosis and inflammatory responses, potentially contributing to melanoma prognosis and therapy [17]. In oral cancer (OC), the expression of RTP4 is significantly elevated in cancer tissues higher than in normal tissues, and it is related to the poor prognosis of OC patients [18]. In ovarian cancer (OV), RTP4 was found to act as an oncogene that supports the proliferation, migration, and invasion abilities of cancer cells [19]. However, the role and function of RTP4 in CRC remain poorly understood.

In this study, we explored the expression pattern, clinical significance and role of RTP4 in CRC. What's more, the relationship between RTP4 expression and the tumour microenvironment (TME) was also investigated. Finally, through a series of wet experiments, we discovered that RTP4 could delay CRC progression by boosting anti‐tumour immunity.

## 2 Materials and Methods

### 2.1 Public Data Collection

The pan‐cancer expression of RTP family members was investigated through the TIMER database [20]. The expression of RTP4 in different human tissues was investigated and analysed using the HPA, GTEx and FANTOM5 databases [21]. To analyse *RTP4* expression at single‐cell levels, the Single Cell Portal database was used [22]. The expression of *RTP4* in CRC cancer and non‐cancer tissues from GSE21510, GSE25071, GSE29638, GSE71187 and GSE87211 databases was investigated using the BEST database [23].

### 2.2 Cell Culture

MC38, CT26, RKO and HCT116 cell lines were purchased from ATCC. Cells were cultured at 37°C in DMEM with 10% FBS.

### 2.3 Survival Analysis

The expression of *RTP4* and clinical prognostic indicators, including overall survival (OS), recurrence‐free survival (RFS) or post‐progression survival (PPS) of CRC patients, was obtained in GSE12945, GSE17538, GSE39582 and GSE41258. The relationship between *RTP4* expression and clinical prognostic indicators of CRC patients was analysed using the Log‐Rank test.

### 2.4 Cell Transduction

Lentivirus CMV containing overexpressed plasmids (vector or RTP4‐FLAG plasmids) or knockdown plasmids (sh*NC* or sh*B2M* plasmids) was constructed to establish relevant MC38 cell lines. The sequence for sh*NC* is 5′‐CACAGGTTGGTGGTGCAAGTGA‐3′ and for sh*B2M* is 5′‐ACGGTGATTCTAATCATCTTAA‐3′ [24]. In addition, vector or RTP4‐overexpressed MC38‐ovalbumin (OVA) cell lines were also established for subsequent CD8^+^ T cell killing assays.

### 2.5 Co‐Expression Genes Analysis

We utilised the LinkedOmics database to identify co‐expressed genes positively related to the expression of *RTP4* in CRC samples from the TCGA database [25]. The heatmap plot of the co‐expressed genes was also generated through the LinkedOmics database. In addition, correlation analysis between *RTP4* expression and immune‐related genes (including *STAT1*, *ISG15*, *IFI44*, *IRF1*, *USP18*, *DDX60*, *IFI35*, *IRF9*, *STAT2*, *B2M* and *DDX58*) was conducted using the TIMER database [20].

### 2.6 Functional Enrichment Analysis

GSEA‐GO, GSEA‐KEGG and GSEA‐Hallmark analysis of RTP4 in CRC was performed through utilising the BEST database. Metascape was also used to perform GO and KEGG analysis of genes that are significantly positively related to RTP4 expression in CRC [26].

### 2.7 Estimation of Infiltrating Immune Cells in CRC

The ESTIMATE, TIMER and MCPcounter algorithms were used to estimate the fractions of immune infiltrates in CRC tissues from GEO and TCGA databases through using the BEST databases.

### 2.8 Estimation of TMB, MSI and Exclusion Score in CRC

The mRNA expression of *RTP4* in TCGA‐COADREAD was downloaded from the TCGA database. The TMB Score of each sample was estimated through the R package ‘maftools’. The MSI Score of each sample was obtained from a previous study [27]. The Exclusion Score of each sample was estimated through the TIDE algorithm [28]. Spearman's correlation analysis was performed to evaluate the relationship between the expression of *RTP4* and TMB/MSI Scores.

### 2.9 Immunoblotting Analysis

Protein lysates of the relevant cell samples, prepared using RIPA buffer supplemented with protease and phosphatase inhibitors, were quantified by BCA. Protein was then denatured at 95°C for 10 min. Subsequently, 10 μg protein was separated by SDS‐PAGE and transferred electrophoretically onto PVDF membranes. Following transfer, membranes were blocked with 5% non‐fat dry milk in Tris‐buffered saline containing Tween‐20 (TBST) for 1 h at room temperature to prevent non‐specific binding. Membranes were then incubated overnight at 4°C with specific primary antibodies targeting the proteins of interest. After extensive washing with TBST to remove unbound antibodies, membranes were probed with corresponding horseradish peroxidase (HRP)‐conjugated secondary antibodies for 1 h at room temperature. Following further washes, immunoreactive bands were visualised using an enhanced chemiluminescence (ECL) substrate and captured digitally. Primary antibodies used in this study are listed in Table S1.

### 2.10 Cell Proliferation Assay

For the cell proliferation assay, the relevant MC38 or CT26 cells were seeded in flat‐bottom 96‐well plates (3000 cells/well). Cell proliferating rate was estimated by Cell Counting Kit‐8 (CCK8) assay (Beyotime, C0038) at 0, 24, 48 and 72 h.

### 2.11 Flow Cytometry Analysis

For analysing infiltrating CD8^+^ T cells in MC38 tumours, tumours were digested with collagenase D and DNase I, and the single cells were then filtered with 70 μm cell strainers and blocked with anti‐mouse CD16/32. Subsequently, cells were stained with cell surface markers, including CD45, CD8, CD44, CD62L, PD‐1, CTLA‐4 or TIM‐3 antibodies. The antibodies used for flow cytometry are listed in Table S1. For intracellular staining of granzyme B (GZMB) and IFN‐γ in CD8^+^T cells, cells were stained with a fixation/permeabilization buffer solution according to the manufacturer's protocol (BD Biosciences). Before IFN‐γ staining, cells were cultured in vitro for 6 h in the presence of 50 ng/mL phorbol 12‐myristate 13‐acetate (MCE, HY‐18739), 1 μg/mL ionomycin (MCE, HY‐13434) and 5 μg/mL Brefeldin A (MCE, HY‐16592). For analysing MHC class I expression in MC38 or CT26 cells, 3 × 10^5^ cultured cells were harvested and stained with antibodies against H‐2Kd or H‐2Kb at 4°C for 30 min. A flow cytometric analysis was performed using CytoFLEX LX, and the data were analysed using FlowJo software V10.

### 2.12 T‐Cell Killing Assay

Splenic OT‐I T cells were magnetically isolated using the MojoSor Mouse CD8^+^ T Cell Isolation Kit (Biolegend, 480008) from OT‐I mice according to the manufacturer's protocol. OT‐I T cells were then co‐cultured with vector or RTP4‐overexpressed MC38‐OVA cells at a ratio of 2:1, 5:1 or 10:1 in RPMI 1640 medium containing 10% FBS and recombinant mouse IL‐2 (20 ng/mL; R&D Systems, 402‐ML‐100). Apoptotic tumour cells were then quantified using Trypan Blue staining (Beyotime, C0011).

### 2.13 Animal Model

Four‐week‐old NOG mice, 6‐week‐old NOG mice, C57BL/6 mice and BALB/c mice were purchased from Charles River. OT‐I mice were purchased from The Jackson Laboratory. MC38 and CT26 cell lines were used to establish subcutaneous CRC tumour models. To explore the effect of RTP4 overexpression on MC38 tumour growth in the presence or absence of a functional TME, MC38 cells were subcutaneously inoculated into either NOG mice (5 × 10^5^ cells per mouse) or C57BL/6J mice (2 × 10^5^ cells per mouse). To investigate whether the RTP4‐mediated tumour‐suppressive function is partially dependent on MHC‐I expression, we established four experimental groups: Vector control, RTP4‐overexpression, Sh‐*NC* control and RTP4‐overexpression+sh‐*B2M*. The corresponding genetically modified MC38 cells were then subcutaneously inoculated into C57BL/6J mice (2 × 10^5^ cells per mouse). To further assess whether RTP4 overexpression affects the efficiency of ICB therapy in CRC, we set up four treatment groups: Vector+IgG isotype control, Vector+α‐PD‐1, RTP4‐overexpression+IgG isotype control and RTP4‐overexpression+α‐PD‐1. The relevant CT26 cells were subcutaneously inoculated into BALB/c mice (5 × 10^5^ cells per mouse). When tumour volumes reached 70–100 mm^3^, mice were intraperitoneally injected with either anti‐mouse PD‐1 antibody (α‐PD‐1; 250 μg per mouse; Bio X Cell, BE0273) or isotype control antibody every 3 days for up to 3 weeks. The mice were sacrificed by cervical dislocation, and the tumours were measured and collected for further experiments. Tumour volume (mm^3^) was calculated using the following formula: tumour width^2^ × length/2. All animal experimentation in the current study was approved by the Animal Ethics Committee of the First Affiliated Hospital of Nanchang University.

### 2.14 Statistical Analysis

The statistical significance was analysed using Kruskal–Wallis, Mann–Whitney‐Wilcoxon test, one‐way unpaired two‐tailed Student's *t*‐test, one‐way ANOVA or two‐way ANOVA as appropriate. *p* < 0.05 was regarded as statistically significant.

## 3 Results

### 3.1 The Expression of RTP4 in Human Normal and Cancer Tissues

**FIGURE 1:**
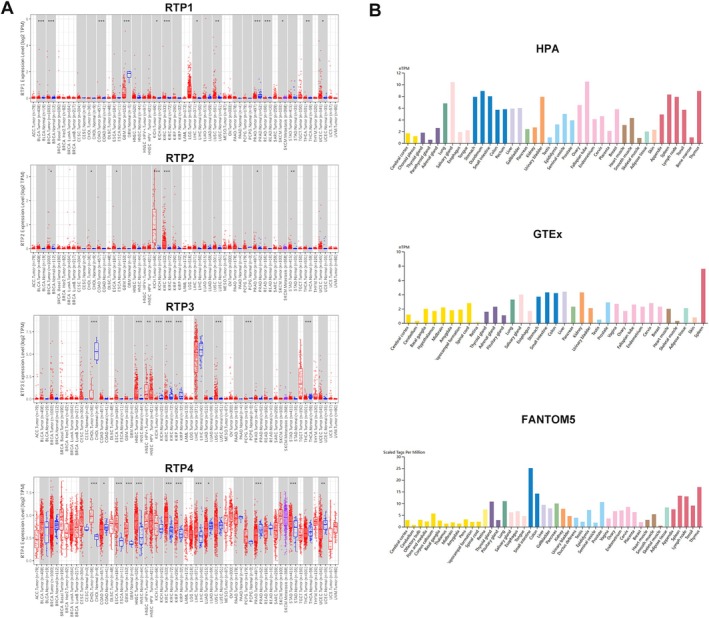
The expression of RTP4 in human normal and cancer tissues. (A) Pan‐cancer analysis of the expression of *RTP1‐4*. (B) The expression of *RTP4* in normal human tissues from the HPA, GTEx and FANTOM5 databases. *p < 0.05, **p < 0.01, ***p < 0.001.

To investigate the potential involvement of RTP family members in tumourigenesis, we first analysed their expression profiles in cancer tissues and paired adjacent noncancerous tissues using data from TCGA. As depicted in Figure S1, *RTP1, RTP2* and *RTP3* exhibited consistently low expression levels in cancer tissues across multiple tumour types, whereas *RTP4* was relatively highly expressed in cancer tissues compared to their non‐cancer counterparts. Notably, RTP3 displayed a striking cancer‐type specificity, with its expression being predominantly associated with hepatocellular carcinoma (HCC) among the analysed cancer types. However, subsequent analysis revealed that *RTP3* expression did not differ significantly between HCC tissues and paired non‐cancer liver tissues (Figure 1A). In contrast, although RTP4 lacked the same degree of cancer‐type specificity, its expression was found to be aberrantly regulated—either significantly upregulated or downregulated—in several distinct cancer types, suggesting a context‐dependent role in tumour pathology. We then further explored the expression of *RTP4* in different normal organ tissues. Compared with other organs, the expression level of *RTP4* is highest in the digestive system organs, especially in the intestine (Figure 1B). Interestingly, we found that the expression level of *RTP4* is significantly lower in CRC than in non‐cancer colon tissues, which suggested that RTP4 may play a key role in the progression of CRC (Figure 1A). Therefore, we chose to further explore the function of RTP4 in CRC.

### 3.2 *RTP4* Expression Is an Independent Predictive Factor for Clinical Outcomes in CRC Patients

**FIGURE 2:**
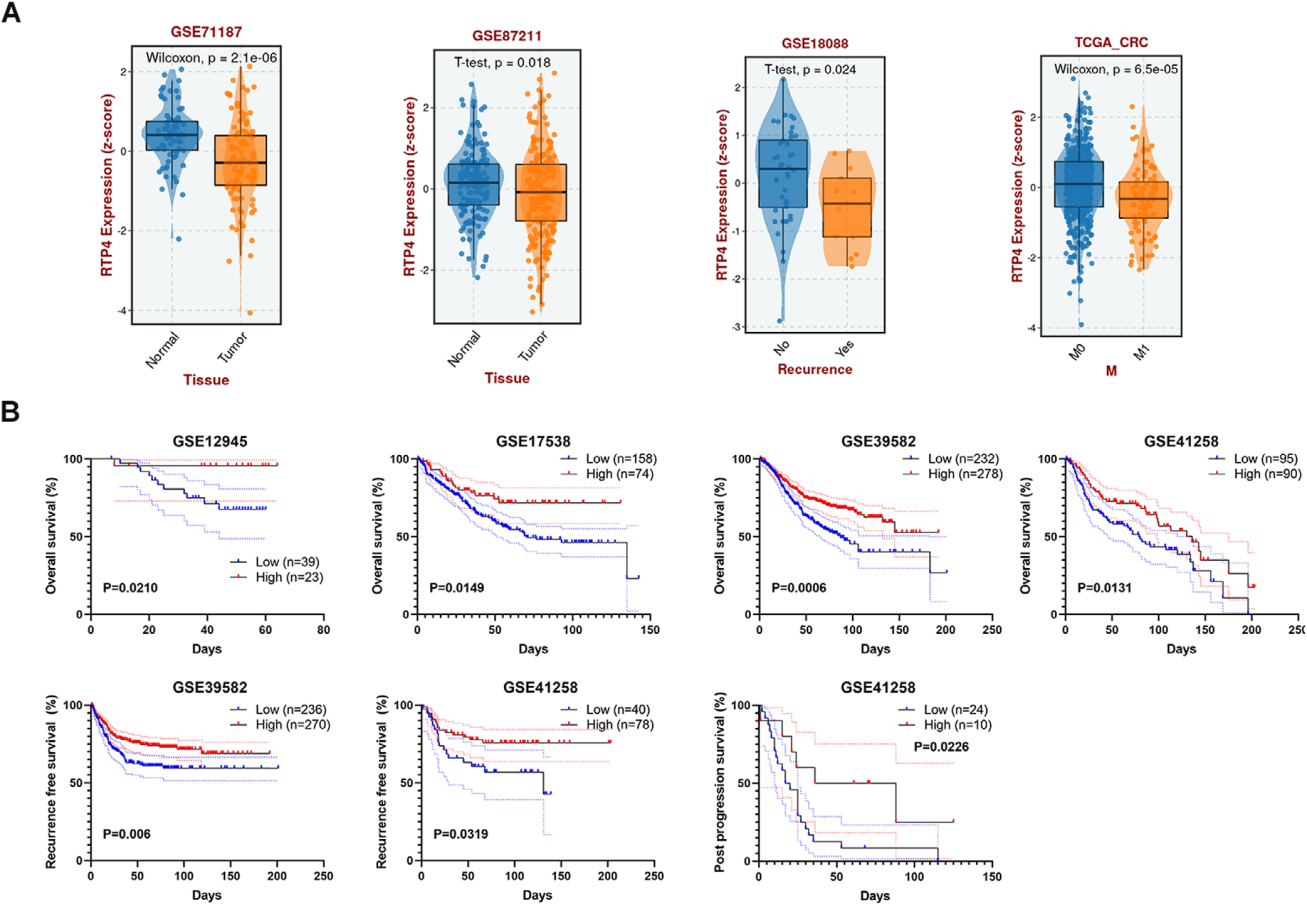
*RTP4* expression patterns and clinical significance in CRC. (A) The expression of *RTP4* in CRC (primary or metastatic) and non‐cancer tissues. (B) The relationship between *RTP4* expression and the overall survival (OS), recurrence‐free survival (RFS) and post‐progression survival (PPS) of CRC patients.

We then further investigated the expression patterns of *RTP4* in public CRC databases using the BEST database. As shown in Figure 2A, the expression of *RTP4* is significantly downregulated in CRC samples, especially in CRC metastasis samples. Subsequently, we explored the relationship between the low expression of *RTP4* and the clinical prognosis of CRC patients. As shown in Figure 2B, we found that the low expression of *RTP4* is markedly related to worse OS, RFS and PPS in CRC patients. This indicates that *RTP4* expression may act as an independent predictive factor for clinical outcomes in CRC patients.

### 3.3 *RTP4* Expression Is Related to the Enhanced Anti‐Tumour Immune Responses

**FIGURE 3:**
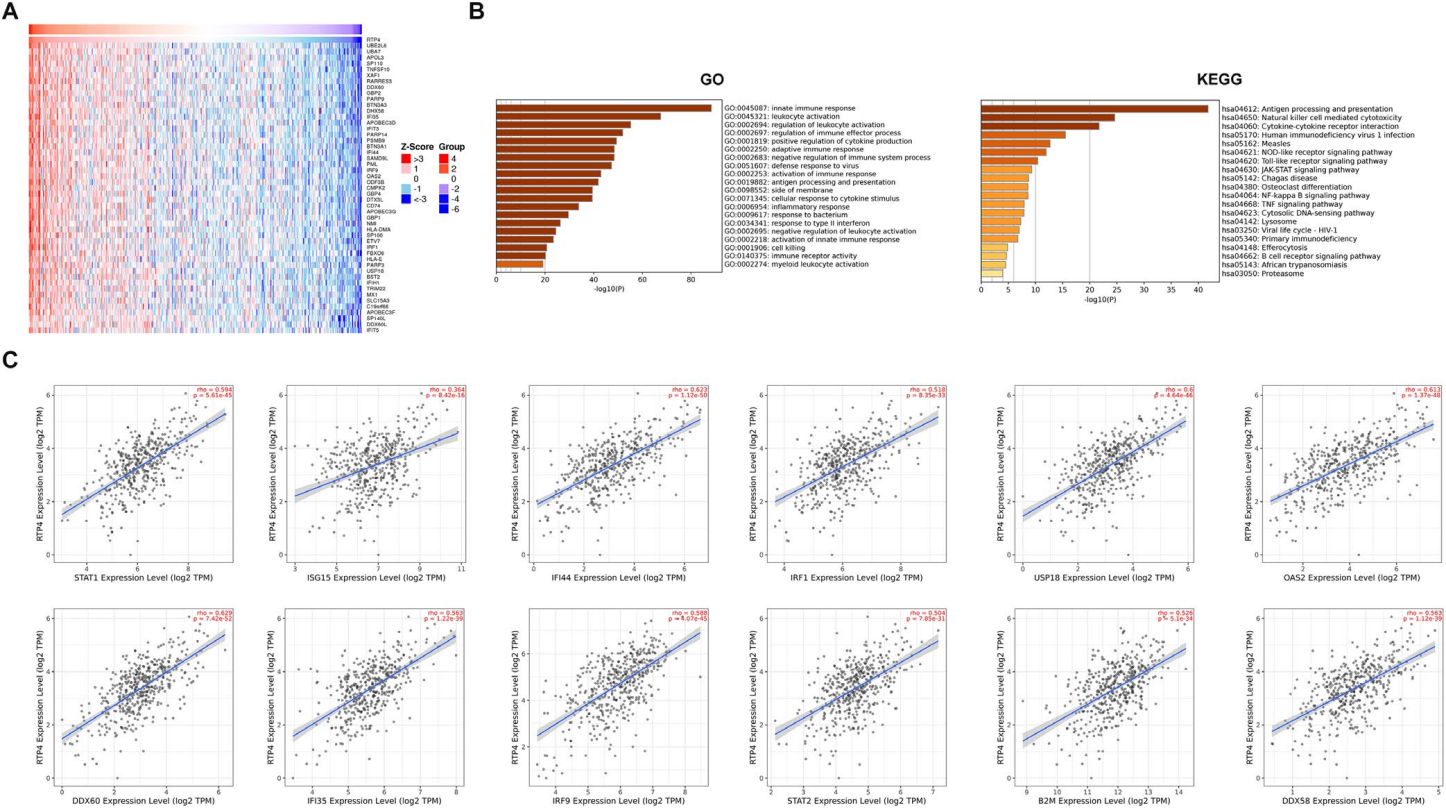
Correlation and functional enrichment analysis of RTP4 in CRC. (A) Genes that are positively related to *RTP4* expression in CRC. (B) GO and KEGG functional enrichment analysis of genes that are positively related to RTP4's expression. (C) Correlation analysis of *RTP4* expression and immune‐related genes such as *STAT1*, *ISG15*, *IFI44*, *IRF1*, *USP18*, *DDX60*, *IFI35*, *IRF9*, *STAT2*, *B2M* and *DDX58*.

To explore the biological roles of RTP4 in CRC, we screened for genes that were markedly positively correlated with the expression of *RTP4* using the LinkedOmics database (Figure 3A). Subsequently, we performed GO and KEGG analyses of these genes. As a result, genes that are positively associated with *RTP4* expression are mostly enriched in items related to the activation of anti‐tumour immune responses, such as innate immune response (GO:0045087), leukocyte activation (GO:0045321), regulation of leukocyte activation (GO:0002694), antigen processing and presentation (hsa04612), Natural killer cell mediated cytotoxicity (hsa04650) and Cytokine‐cytokine receptor interaction (hsa04060) (Figure 3B). Correlation analysis also confirmed that *RTP4* expression is significantly related to the expression of several immune‐related genes, such as *STAT1*, *ISG15*, *IFI44*, *IRF1*, *USP18*, *DDX60*, *IFI35*, *IRF9*, *STAT2*, *B2M* and *DDX58* (Figure 3C).

Subsequently, we also performed GSEA‐GO, GSEA‐KEGG and GSEA‐Hallmark analysis of RTP4 in CRC. As a result, we found that RTP4 expression is positively related to items regarding the enhanced anti‐tumour immune responses, such as antigen processing and presentation of peptide antigen, adaptive immune response, antigen processing and presentation, cytokine cytokine receptor interaction, T cell receptor signalling pathway, complement, inflammatory response and interferon alpha response (Figure S2). These results suggested that RTP4 may contribute to the enhanced immune responses in CRC.

### 3.4 *RTP4* Expression Is Correlated to Immune Infiltrates in CRC and Related to Immunotherapy Responses

**FIGURE 4:**
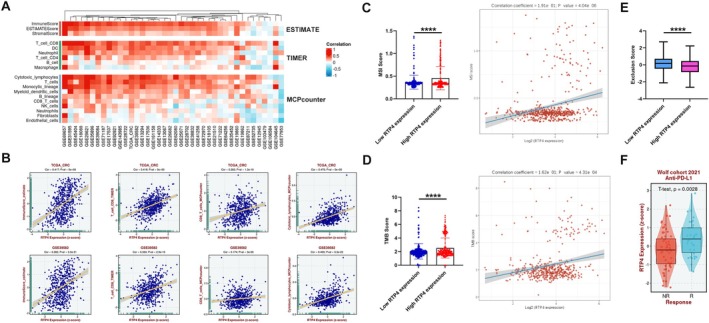
The association of *RTP4* expression and TME in CRC. (A) The ESTIMATE, TIMER and MCPcounter algorithms were used to estimate the fractions of immune infiltrates and their relationship with *RTP4* expression in CRC tissues. (B) Correlation analysis of *RTP4* expression and the fraction of infiltrated immune cells. (C–E) The relationship between RTP4's expression and (C) MSI score, (D) TMB score and (E) Exclusion score of samples from the TCGA‐COADREAD database. (F) The expression of *RTP4* in non‐responders/responders that received anti‐PD‐L1 treatment in the Wolf cohort.

First, we used the ESTIMATE, TIMER and MCPcounter algorithms to estimate the fractions of immune infiltrates in CRC tissues from TCGA and several GEO databases. Through the ESTIMATE algorithm, we found that *RTP4* expression is positively related to ImmunScore, ESTIMATEScore and StromalScore in TCGA and most of the relevant GEO databases (Figure 4A,B). Through the TIMER algorithm, we found that *RTP4* expression is positively associated with infiltrated CD8+T cells in TCGA and most of the relevant GEO databases (Figure 4A,B). Through the MCPcounter algorithm, we found that *RTP4* expression is positively associated with infiltrated cytotoxic lymphocytes and T cells in TCGA and most of the relevant GEO databases (Figure 4A,B). These results suggested that *RTP4* is related to the increased infiltration of anti‐tumour immune cells, especially anti‐tumour CD8^+^T cells.

To explore the association of RTP4 and CRC immunotherapy responses, we then estimated the MSI score, TMB score and Exclusion score of patients from the TCGA‐COADREAD database. As a result, we found that samples with high *RTP4* expression had higher MSI or TMB scores than samples with low RTP4 expression. The expression of *RTP4* was also significantly positively related to MSI or TMB scores (Figure 4C,D). Due to cancer patients with high TMB or MSI status having a higher response efficiency to immunotherapy than patients with low TMB or MSS status, the expression of *RTP4* may act as a biomarker for predicting immunotherapy responses. In addition to MSI or TMB scores, we also estimated the Exclusion score. As a result, we found that samples with high *RTP4* expression had a lower Exclusion score than samples with low *RTP4* expression (Figure 4E). In addition, in a clinical cohort that contains cancer patients who received anti‐PD‐L1 treatment, *RTP4's* expression is significantly lower in non‐responders than in responders (Figure 4F). These results indicate that high *RTP4* expression may be related to enhanced immunotherapy responses in CRC patients.

### 3.5 RTP4 Overexpression Inhibits CRC Tumour Growth via Promoting CD8 ^+^T Cell Infiltration

**FIGURE 5:**
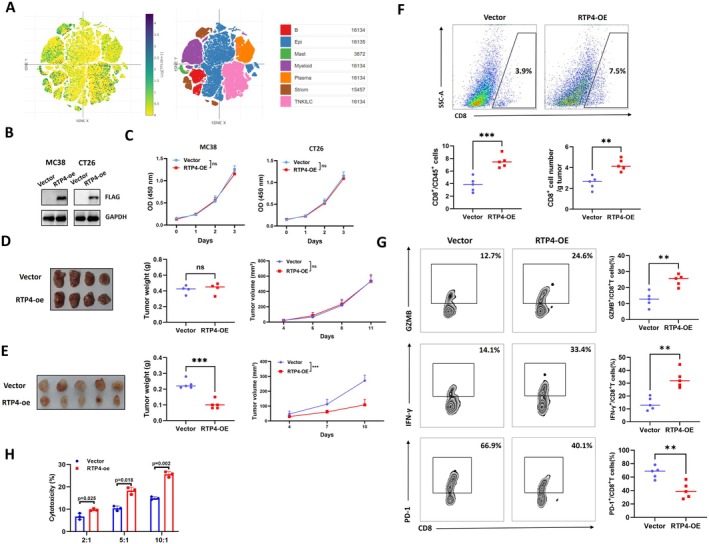
RTP4 overexpression delays CRC progression via enhancing anti‐tumour CD8^+^T cell immunity. (A) Single‐cell analysis of the expression of *RTP4* in human CRC samples. (B) WB analysis confirmed the stable overexpression of RTP4 in MC38 and CT26 cells via detecting the FLAG tag. (C) CCK8 analysis of vector and RTP4‐overexpressed MC38 and CT26 cells. (D) The effect of RTP4 overexpression on the MC38 tumour growth in NOG mice. (E) The effect of RTP4 overexpression on the MC38 tumour growth in C57BL/6J mice. (F) The fraction of tumour‐infiltrating CD8^+^ T cells in MC38 tumours was analysed using flow cytometry, gated on CD45^+^ cells. The absolute numbers of CD8^+^ T cells were also quantified. (G) Flow cytometry was also performed to assess the proportions of activated (IFN‐γ^+^/GZMB^+^) and exhausted (PD‐1^+^) subsets within CD8^+^ T cells. (H) Co‐culture of vector and RTP4‐overexpressed MC38‐OVA with CD8^+^ T cells from OT‐I mice. (*n* = 3 biologically independent samples per group). Two‐tailed unpaired Student's *t*‐test was used.

Single‐cell RNA sequencing (scRNA‐seq), a pivotal technology for delineating gene expression heterogeneity across diverse cell types within the TME, was employed to investigate *RTP4's* expression in CRC. As illustrated in Figure 5A, *RTP4's* expression was detected in both cancer cells and immune cell populations within the CRC TME. Based on this observation, we next sought to determine whether RTP4 overexpression in tumour cells modulates CRC progression. We first constructed RTP4‐overexpressed MC38 and CT26 CRC cell lines. WB analysis confirmed the successful overexpression of RTP4‐FLAG (Figure 5B). Subsequently, we explored the effect of RTP4 on MC38 and CT26 cell proliferation ability in vitro. As shown in Figure 5C, we found that RTP4 overexpression did not affect the proliferation ability of MC38 and CT26 cells.

To further explore the effects of RTP4 on CRC tumour growth in vivo, we injected vector or RTP4‐overexpressed MC38 cells subcutaneously into NOG (without lymphocytes) and C57BL/6J (with lymphocytes) mice, respectively. Interestingly, we found that there was no significant difference in the tumour growth rate and tumour weight between the vector and RTP4‐overexpressed MC38 tumours in NOG mice (Figure 5D). However, the tumour growth rate of RTP4‐overexpressed MC38 tumours was significantly lower and the tumour weight was significantly smaller than that of vector‐expressed MC38 tumours in C57BL/6J mice (Figure 5E). These results indicate that RTP4 can delay CRC progression only by modulating anti‐tumour immunity. To verify this, we performed a flow cytometry analysis of the two groups of MC38 tumours. As a result, we found that the fraction and absolute numbers of total infiltrating CD8^+^ T cells were significantly higher in RTP4‐overexpressed MC38 tumours (Figure 5F). Subsequently, we detected the fractions of naïve (CD44^−^CD62L^+^), central memory (CD44^+^CD62L^+^) and effector (CD44^+^CD62L^−^) CD8^+^ T cells as well as functional (IFN‐γ^+^/GZMB^+^) and exhausted (PD‐1^+^/TIM‐3^+^/CTLA‐4^+^) CD8^+^ T cells. As shown in Figure S3, the fraction of effector CD8^+^ T cells was significantly increased in RTP4‐overexpressed MC38 tumours, while there were no significant changes in naïve T cells or central memory T cells. In addition, the fractions of GZMB^+^/IFN‐γ^+^ CD8^+^ T cells were markedly increased in RTP4‐overexpressed MC38 tumours, while PD‐1^+^/TIM‐3^+^/CTLA‐4^+^ CD8^+^T cells were markedly decreased (Figure 5G, Figures S3 and S4). To further validate whether RTP4 affects anti‐tumour immunity through regulating CD8^+^ T cell activity, vector or RTP4‐overexpressed MC38 cells were co‐cultured with CD8^+^ T cells isolated from C57BL/6J mice. As shown in Figure 5H, CD8^+^ T cells co‐cultured with RTP4‐overexpressed MC38 cells at a ratio of 10:1 showed higher cytotoxic activities than those co‐cultured with vector MC38 cells. These results confirmed that RTP4 could inhibit CRC tumour growth by enhancing CD8^+^ T cell infiltration and promoting their tumour cell‐killing function.

### 3.6 RTP4 Inhibits CRC Immune Evasion via Promoting MHC‐I Expression

**FIGURE 6:**
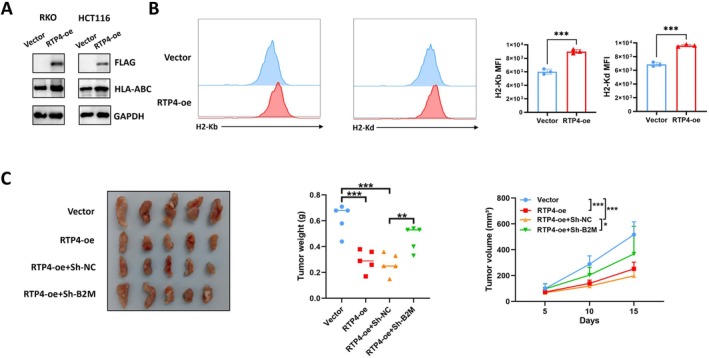
RTP4 overexpression boosts anti‐tumour immunity by increasing MHC‐I expression. (A) WB analysis of MHC‐I expression in vector and RTP4‐overexpressed RKO and HCT116 cells. (B) Flow cytometry analysis of H‐2Kb or H‐2Kd expression on vector and RTP4‐overexpressed MC38 and CT26 cells. (C) The effect of RTP4 overexpression with/without *B2M* knockdown on the MC38 tumour growth in C57BL/6J mice. *p < 0.05, **p < 0.01, ***p < 0.001, ns, no significance, by two‐tailed unpaired Student's *t*‐test or one‐way ANOVA except analysis of tumour volume in: two‐way ANOVA.

We then further explore the mechanism of RTP4 that promotes anti‐tumour CD8^+^T cell immunity in CRC. According to the previous results of GSEA analysis of *RTP4*, we found that *RTP4* expression is positively associated with antigen processing and presentation of peptide antigens, a process critically dependent on MHC‐I expression on the tumour cell surface. Thus, we explored whether RTP4 may regulate MHC‐I expression on CRC cells. We then constructed RTP4‐overexpressed human CRC RKO and HCT116 cell lines (Figure 6A). As shown in Figure 6B, MHC‐I expression is significantly upregulated in RTP4‐overexpressed RKO and HCT116 cells, and the same results were also discovered in MC38 and CT26 cells. Next, we confirmed that the RTP4‐mediated tumour‐suppressive function was partially dependent on *B2M*, as *B2M* knockdown partially impaired the growth‐inhibitory effect of RTP4 overexpression on MC38 tumours (Figure 6C).

### 3.7 RTP4 Overexpression Increases CRC ICB Treatment Responses

**FIGURE 7:**
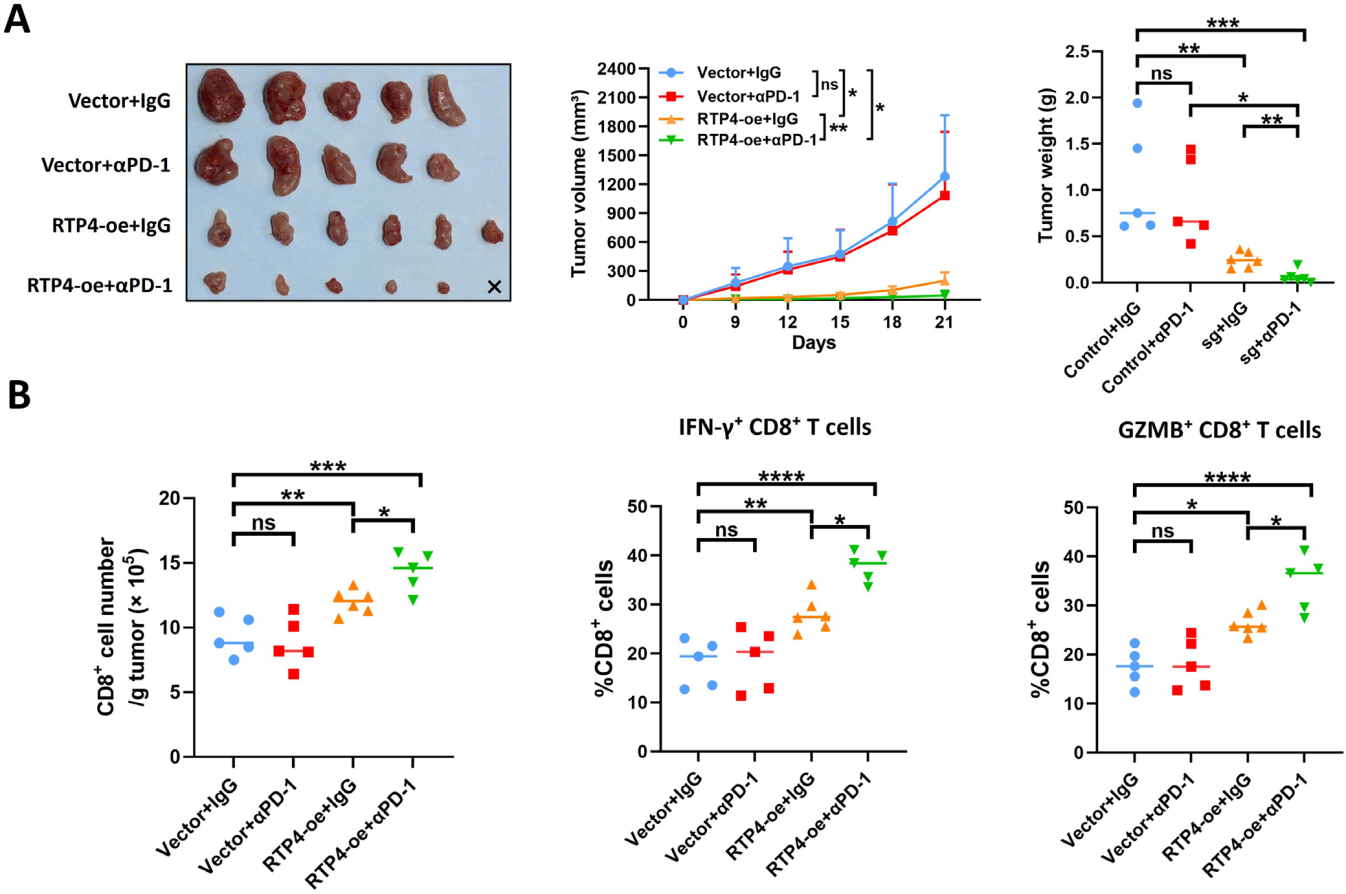
RTP4 overexpression increases ICB treatment responses in CT26 tumours. (A) The growth of vector and RTP4‐overexpressed CT26 tumours received IgG or anti–PD–1 antibody treatment. (B) Flow cytometry was performed to analyse tumour‐infiltrating CD8^+^ T cells in CT26 tumours, including total CD8^+^ T cells, activated (IFN‐γ^+^/GZMB^+^) CD8^+^ T cells, and exhausted (PD‐1^+^) CD8^+^ T cells. The absolute numbers of CD8^+^ T cells were quantified, and the proportions of IFN‐γ^+^, GZMB^+^ and PD‐1^+^ subsets within CD8^+^ T cells were also assessed. *p < 0.05, **p < 0.01, ***p < 0.001, ****p < 0.0001, ns, no significance, by one‐way ANOVA except analysis of tumour volume in (A): two‐way ANOVA.

To discover whether the overexpression of RTP4 can affect the ICB treatment responses in CRC, we treated with anti–PD‐1 antibody or isotype antibody in Balb/c mice bearing CT26 tumours. As shown in Figure 7A, the overexpression of RTP4 in CT26 cells could significantly improve the anti‐tumour effects of ICB treatment. In addition, the RTP4‐overexpressed CT26 tumours that received ICB treatment had the highest fraction of infiltrating total CD8^+^ T and GZMB^+^/IFN‐γ^+^ CD8^+^ T cells (Figure 7B). These results suggest that RTP4 overexpression may improve ICB treatment responses in CRC.

## 4 Discussion

RTP4, as one of the members of the RTP family, has been found to be associated with the prognosis of cancer patients and may regulate cancer progression [15, 16, 17, 18, 19]. However, the role of RTP4 in CRC has not been well explored. In the present study, we found that the expression of *RTP4* is significantly downregulated in CRC. In addition, the downregulation of *RTP4* is significantly associated with the worse clinical prognosis of CRC patients, indicating it may act as a novel prognostic biomarker in CRC.

Through co‐expression gene analysis, we found that *RTP4* expression is significantly positively related to immune‐related genes such as *STAT1*, *ISG15*, *IFI44*, *IRF1*, *USP18*, *DDX60*, *IFI35*, *IRF9*, *STAT2*, *B2M* and *DDX58*. Interestingly, we found that most of these genes are related to the type I interferon (IFN) signalling. In both normal and cancer cells, upon the stimulation of type I IFNs such as IFN‐α and β, STAT1 and STAT2 can be phosphorylated, leading to heterodimerization and interaction with IRF9 [29, 30]. This complex then translocates into the nucleus and leads to the activation of interferon‐stimulated genes (ISGs), such as *ISG15*, *IFI44*, *USP18* and *DDX60* [31, 32, 33, 34]. *IFI35* is another ISG and was found to promote the proliferation and cytotoxic activity of CD8^+^T cells in CRC through regulating the PI3K/AKT/mTOR axis [35]. *B2M* encodes the β chain of MHC class I molecules and is a key component in MHC‐I mediated antigen processing and presentation, which promotes the recruitment and activation of anti‐tumour CD8^+^ T cells [36]. *DDX58* encodes retinoic acid‐inducible gene‐I (RIG‐I), activation of which can boost cancer immunotherapy efficiency [37]. These indicate that the upregulation of RTP4 may be related to the enhanced anti‐tumour immunity in CRC, and it has been verified through the subsequent functional enrichment analysis.

To further explore the relationship between RTP4 and the TME, we estimated the fraction of immune infiltrates in CRC and found that *RTP4* expression is significantly related to the higher fraction of infiltrating cytotoxic CD8^+^ T cells. In addition, we found that CRC patients with high *RTP4* expression had higher TMB and MSI scores and lower exclusion scores. This suggested that this part of CRC patients may benefit from ICB treatment.

To verify the function of RTP4 in CRC, we constructed RTP4‐overexpressed MC38 and CT26 cells and performed a series of experiments. Interestingly, we found that RTP4 overexpression did not regulate the proliferation ability of CRC cells. However, we discovered that RTP4 overexpression could delay CRC progression by promoting anti‐tumour immunity. We found that RTP4 overexpression could promote the infiltration of CD8^+^ T cells. In particular, the fraction of effector and functional CD8+ T cells in RTP4‐overexpressed tumours was significantly increased, while the fraction of exhausted CD8^+^ T cells was decreased. These results suggested that RTP4 may inhibit CRC progression through regulating CD8^+^ T cell functions.

The MHC‐I complexes, which are composed of a polymorphic heavy chain and the light chain β2‐microglobulin (β2M), play critical roles in anti‐tumour immunity. These complexes mediate antigen presentation by binding antigenic peptides on the cell surface, enabling recognition by T‐cell receptors (TCRs) on CD8^+^ T cells and subsequent tumour cell elimination [38]. The downregulation of major MHC‐I molecules could be strategically exploited by malignant cells as an immune evasion mechanism, thereby impairing the efficacy of ICB treatment and promoting cancer development [39]. In our study, we revealed that enforced RTP4 expression in CRC cells substantially enhanced MHC‐I expression, whereas knockdown of *B2M* rescued tumour growth inhibition induced by RTP4 overexpression, suggesting that β2M‐dependent immunoregulation underpins this phenotype.

Given the potential association between RTP4 expression and ICB efficacy in CRC, we employed a syngeneic CT26 tumour‐bearing mouse model to investigate the impact of RTP4 overexpression on ICB therapeutic outcomes. The CT26 cell line is classified as proficient mismatch repair/microsatellite stable (pMMR/MSS), a subtype widely reported to exhibit poor responsiveness to ICB treatment [40, 41, 42, 43, 44]. Our experimental results demonstrated minimal tumour growth inhibition in CT26 tumours following anti‐PD‐1 monotherapy. However, RTP4 overexpression significantly enhanced the anti‐tumour efficacy of ICB, which was mechanistically linked to increased infiltration of cytotoxic CD8^+^ T cells expressing GZMB^+^ or IFN‐γ^+^ within the TME. Notably, some studies have reported CT26 responsiveness to ICB [45, 46]. After comparing key treatment parameters (including αPD‐1 dosage, administration frequency and time of first treatment), we observed no significant methodological differences that could explain this discrepancy. Future studies are required to elucidate the underlying mechanisms driving these divergent responses.

## 5 Conclusions

In conclusion, this study demonstrates that RTP4 exerts tumour‐suppressive effects in CRC by upregulating MHC‐I expression, thereby activating CD8^+^T cell‐mediated anti‐tumour immunity. These findings provide mechanistic insights into how RTP4‐mediated immune modulation delays CRC progression. Importantly, our data suggest that therapeutic targeting of RTP4 may synergize with ICB therapy, potentially overcoming current limitations in CRC immunotherapeutic efficacy.

## Author Contributions

**Chengpeng Yu:** formal analysis (equal), investigation (equal), software (equal), supervision (equal), writing – original draft (equal), writing – review and editing (equal). **Yifan Li:** investigation (equal), software (equal). **Zhenzhe Liu:** investigation (equal), methodology (equal). **Wen Ye:** validation (equal), visualization (equal). **Lin Wei:** investigation (equal). **Weiqi Xu:** validation (equal). **Likun Yan:** formal analysis (equal). **Haifeng Ye:** funding acquisition (equal), project administration (equal), writing – review and editing (equal).

## Consent

The authors have nothing to report.

## Conflicts of Interest

The authors declare no conflicts of interest.

## Supporting information


**Figure S1:** The expression levels of *RTP1*, *RTP2*, *RTP3* and *RTP4* in cancer tissues across multiple tumour types.

**Figure S2:** GSEA analysis of RTP4 in CRC. (A) GSEA‐GO analysis of RTP4. (B) GSEA‐KEGG analysis of RTP4. (C) GSEA‐Hallmark analysis of RTP4.

**Figure S3:** Flow cytometry was performed to analyse the fractions of naïve (CD44^−^CD62L^+^), central memory (CD44^+^CD62L^+^) and effector (CD44^+^CD62L^−^) CD8^+^ T cells in MC38 tumours. *p < 0.05, ns, no significance, by two‐tailed unpaired Student's *t*‐test.

**Figure S4:** Flow cytometry was performed to analyse the fractions of TIM‐3^+^ or CTLA‐4^+^ CD8^+^ T cells in MC38 tumours. *p < 0.05, ns, no significance, by two‐tailed unpaired Student's *t*‐test.

**Table S1:** Antibodies used in this study.

## Data Availability

All analysed datasets are available in the HPA, GTEx, FANTOM5, TCGA, GEO, the Single Cell Portal and the BEST databases.

## References

[1] R. L. Siegel , K. D. Miller , A. Goding Sauer , et al., “Colorectal Cancer Statistics, 2020,” CA: A Cancer Journal for Clinicians 70, no. 3 (2020): 145–164. 32133645 10.3322/caac.21601

[2] R. L. Siegel , N. S. Wagle , A. Cercek , et al., “Colorectal Cancer Statistics, 2023,” CA: A Cancer Journal for Clinicians 73, no. 3 (2023): 233–254. 36856579 10.3322/caac.21772

[3] L. H. Biller and D. Schrag , “Diagnosis and Treatment of Metastatic Colorectal Cancer: A Review,” JAMA 325, no. 7 (2021): 669–685. 33591350 10.1001/jama.2021.0106

[4] J. Aparicio , F. Esposito , S. Serrano , et al., “Metastatic Colorectal Cancer. First Line Therapy for Unresectable Disease,” Journal of Clinical Medicine 9, no. 12 (2020): 3889. 33265959 10.3390/jcm9123889 PMC7761096

[5] K. Ganesh , Z. K. Stadler , A. Cercek , et al., “Immunotherapy in Colorectal Cancer: Rationale, Challenges and Potential,” Nature Reviews Gastroenterology & Hepatology 16, no. 6 (2019): 361–375. 30886395 10.1038/s41575-019-0126-x PMC7295073

[6] Z. Jin and F. A. Sinicrope , “Mismatch Repair‐Deficient Colorectal Cancer: Building on Checkpoint Blockade,” Journal of Clinical Oncology: Official Journal of the American Society of Clinical Oncology 40, no. 24 (2022): 2735–2750. 35649217 10.1200/JCO.21.02691 PMC9390830

[7] D. Y. Lizardo , C. Kuang , S. Hao , et al., “Immunotherapy Efficacy on Mismatch Repair‐Deficient Colorectal Cancer: From Bench to Bedside,” Biochimica et Biophysica Acta, Reviews on Cancer 1874, no. 2 (2020): 188447. 33035640 10.1016/j.bbcan.2020.188447 PMC7886024

[8] R. Cohen , B. Rousseau , J. Vidal , R. Colle , L. A. Diaz, Jr. , and T. André , “Immune Checkpoint Inhibition in Colorectal Cancer: Microsatellite Instability and Beyond,” Targeted Oncology 15, no. 1 (2020): 11–24. 31786718 10.1007/s11523-019-00690-0

[9] Z. Bai , Y. Zhou , Z. Ye , J. Xiong , H. Lan , and F. Wang , “Tumor‐Infiltrating Lymphocytes in Colorectal Cancer: The Fundamental Indication and Application on Immunotherapy,” Frontiers in Immunology 12 (2021): 808964. 35095898 10.3389/fimmu.2021.808964 PMC8795622

[10] J. Mainland and H. Matsunami , “RAMP Like Proteins: RTP and REEP Family of Proteins,” Advances in Experimental Medicine and Biology 744 (2012): 75–86. 22434109 10.1007/978-1-4614-2364-5_7

[11] M. Behrens , J. Bartelt , C. Reichling , et al., “Members of RTP and REEP Gene Families Influence Functional Bitter Taste Receptor Expression,” Journal of Biological Chemistry 281, no. 29 (2006): 20650–20659. 16720576 10.1074/jbc.M513637200

[12] F. M. Décaillot , R. Rozenfeld , A. Gupta , and L. A. Devi , “Cell Surface Targeting of Mu‐Delta Opioid Receptor Heterodimers by RTP4,” Proceedings of the National Academy of Sciences of the United States of America 105, no. 41 (2008): 16045–16050. 18836069 10.1073/pnas.0804106105 PMC2572960

[13] J. W. Schoggins , S. J. Wilson , M. Panis , et al., “A Diverse Range of Gene Products Are Effectors of the Type I Interferon Antiviral Response,” Nature 472, no. 7344 (2011): 481–485. 21478870 10.1038/nature09907 PMC3409588

[14] C. Hoyo‐Becerra , Z. Liu , J. Yao , et al., “Rapid Regulation of Depression‐Associated Genes in a New Mouse Model Mimicking Interferon‐α‐Related Depression in Hepatitis C Virus Infection,” Molecular Neurobiology 52, no. 1 (2015): 318–329. 25159480 10.1007/s12035-014-8861-z

[15] N. Xu , Y. P. Wu , Z. B. Ke , et al., “Identification of Key DNA Methylation‐Driven Genes in Prostate Adenocarcinoma: An Integrative Analysis of TCGA Methylation Data,” Journal of Translational Medicine 17, no. 1 (2019): 311. 31533842 10.1186/s12967-019-2065-2 PMC6751626

[16] Y. Li , J. Qi , and J. Yang , “RTP4 Is a Novel Prognosis‐Related Hub Gene in Cutaneous Melanoma,” Hereditas 158, no. 1 (2021): 22. 34154655 10.1186/s41065-021-00183-z PMC8215788

[17] Y. Xu , Y. Chen , Z. Niu , et al., “A Novel Pyroptotic and Inflammatory Gene Signature Predicts the Prognosis of Cutaneous Melanoma and the Effect of Anticancer Therapies,” Frontiers in Medicine 9 (2022): 841568. 35492358 10.3389/fmed.2022.841568 PMC9053829

[18] A. Reyimu , Y. Chen , X. Song , W. Zhou , J. Dai , and F. Jiang , “Identification of Latent Biomarkers in Connection With Progression and Prognosis in Oral Cancer by Comprehensive Bioinformatics Analysis,” World Journal of Surgical Oncology 19, no. 1 (2021): 240. 34384424 10.1186/s12957-021-02360-w PMC8361649

[19] C. Fang , W. Han , C. Tang , J. Shen , and B. Ni , “Identification of RTP4 Facilitating Ovarian Cancer by Bioinformatics Analysis and Experimental Validation,” Naunyn‐Schmiedeberg's Archives of Pharmacology 398, no. 3 (2025): 2665–2675. 39249504 10.1007/s00210-024-03421-z

[20] T. Li , J. Fu , Z. Zeng , et al., “TIMER2.0 for Analysis of Tumor‐Infiltrating Immune Cells,” Nucleic Acids Research 48, no. W1 (2020): W509–W514. 32442275 10.1093/nar/gkaa407 PMC7319575

[21] M. Uhlén , L. Fagerberg , B. M. Hallström , et al., “Proteomics. Tissue‐Based Map of the Human Proteome,” Science (New York, N.Y.) 347, no. 6220 (2015): 1260419. 25613900 10.1126/science.1260419

[22] K. Pelka , M. Hofree , J. H. Chen , et al., “Spatially Organized Multicellular Immune Hubs in Human Colorectal Cancer,” Cell 184, no. 18 (2021): 4734–4752.e20. 34450029 10.1016/j.cell.2021.08.003 PMC8772395

[23] Z. Liu , L. Liu , S. Weng , et al., “BEST: A Web Application for Comprehensive Biomarker Exploration on Large‐Scale Data in Solid Tumors,” Journal of Big Data 10, no. 1 (2023): 165.

[24] Y. Zhao , Y. Cao , Y. Chen , et al., “B2M Gene Expression Shapes the Immune Landscape of Lung Adenocarcinoma and Determines the Response to Immunotherapy,” Immunology 164, no. 3 (2021): 507–523. 34115389 10.1111/imm.13384 PMC8517590

[25] S. V. Vasaikar , P. Straub , J. Wang , and B. Zhang , “LinkedOmics: Analyzing Multi‐Omics Data Within and Across 32 Cancer Types,” Nucleic Acids Research 46, no. D1 (2018): D956–D963. 29136207 10.1093/nar/gkx1090 PMC5753188

[26] Y. Zhou , B. Zhou , L. Pache , et al., “Metascape Provides a Biologist‐Oriented Resource for the Analysis of Systems‐Level Datasets,” Nature Communications 10, no. 1 (2019): 1523. 10.1038/s41467-019-09234-6 PMC6447622 30944313

[27] R. Bonneville , M. A. Krook , E. A. Kautto , et al., “Landscape of Microsatellite Instability Across 39 Cancer Types,” JCO Precision Oncology 2017 (2017): PO.17.00073. 29850653 10.1200/PO.17.00073 PMC5972025

[28] P. Jiang , S. Gu , D. Pan , et al., “Signatures of T Cell Dysfunction and Exclusion Predict Cancer Immunotherapy Response,” Nature Medicine 24, no. 10 (2018): 1550–1558. 10.1038/s41591-018-0136-1 PMC6487502 30127393

[29] L. Zitvogel , L. Galluzzi , O. Kepp , M. J. Smyth , and G. Kroemer , “Type I Interferons in Anticancer Immunity,” Nature Reviews Immunology 15, no. 7 (2015): 405–414. 10.1038/nri3845 26027717

[30] K. Chen , J. Liu , and X. Cao , “Regulation of Type I Interferon Signaling in Immunity and Inflammation: A Comprehensive Review,” Journal of Autoimmunity 83 (2017): 1–11. 28330758 10.1016/j.jaut.2017.03.008

[31] H. M. Nguyen , S. Gaikwad , M. Oladejo , M. Y. Agrawal , S. K. Srivastava , and L. M. Wood , “Interferon Stimulated Gene 15 (ISG15) in Cancer: An Update,” Cancer Letters 556 (2023): 216080. 36736853 10.1016/j.canlet.2023.216080

[32] W. M. Schneider , M. D. Chevillotte , and C. M. Rice , “Interferon‐Stimulated Genes: A Complex Web of Host Defenses,” Annual Review of Immunology 32 (2014): 513–545. 10.1146/annurev-immunol-032713-120231 PMC4313732 24555472

[33] A. Basters , K. P. Knobeloch , and G. Fritz , “USP18 – A Multifunctional Component in the Interferon Response,” Bioscience Reports 38, no. 6 (2018): BSR20180250. 30126853 10.1042/BSR20180250 PMC6240716

[34] D. Goubau , A. G. Van Der Veen , P. Chakravarty , et al., “Mouse Superkiller‐2‐Like Helicase DDX60 Is Dispensable for Type I IFN Induction and Immunity to Multiple Viruses,” European Journal of Immunology 45, no. 12 (2015): 3386–3403. 26457795 10.1002/eji.201545794 PMC4833184

[35] P. Li , D. Zhou , D. Chen , et al., “Tumor‐Secreted IFI35 Promotes Proliferation and Cytotoxic Activity of CD8(+) T Cells Through PI3K/AKT/mTOR Signaling Pathway in Colorectal Cancer,” Journal of Biomedical Science 30, no. 1 (2023): 47. 37380972 10.1186/s12929-023-00930-6 PMC10303345

[36] H. Wang , B. Liu , and J. Wei , “Beta2‐Microglobulin (B2M) in Cancer Immunotherapies: Biological Function, Resistance and Remedy,” Cancer Letters 517 (2021): 96–104. 34129878 10.1016/j.canlet.2021.06.008

[37] Y. Jiang , H. Zhang , J. Wang , et al., “Exploiting RIG‐I‐Like Receptor Pathway for Cancer Immunotherapy,” Journal of Hematology & Oncology 16, no. 1 (2023): 8. 36755342 10.1186/s13045-023-01405-9 PMC9906624

[38] X. Wu , T. Li , R. Jiang , X. Yang , H. Guo , and R. Yang , “Targeting MHC‐I Molecules for Cancer: Function, Mechanism, and Therapeutic Prospects,” Molecular Cancer 22, no. 1 (2023): 194. 38041084 10.1186/s12943-023-01899-4 PMC10693139

[39] J. Wang , Q. Lu , X. Chen , et al., “Targeting MHC‐I Inhibitory Pathways for Cancer Immunotherapy,” Trends in Immunology 45, no. 3 (2024): 177–187. 38433029 10.1016/j.it.2024.01.009 PMC12117934

[40] G. Germano , S. Lamba , G. Rospo , et al., “Inactivation of DNA Repair Triggers Neoantigen Generation and Impairs Tumour Growth,” Nature 552, no. 7683 (2017): 116–120. 29186113 10.1038/nature24673

[41] R. Mandal , R. M. Samstein , K. W. Lee , et al., “Genetic Diversity of Tumors With Mismatch Repair Deficiency Influences Anti‐PD‐1 Immunotherapy Response,” Science (New York, N.Y.) 364, no. 6439 (2019): 485–491. 31048490 10.1126/science.aau0447 PMC6685207

[42] L. Nebot‐Bral , A. Hollebecque , A. A. Yurchenko , et al., “Overcoming Resistance to αPD‐1 of MMR‐Deficient Tumors With High Tumor‐Induced Neutrophils Levels by Combination of αCTLA‐4 and αPD‐1 Blockers,” Journal for ImmunoTherapy of Cancer 10, no. 7 (2022): e005059. 35896284 10.1136/jitc-2022-005059 PMC9335020

[43] Y. Bao , J. Zhai , H. Chen , et al., “Targeting m^6^A Reader YTHDF1 Augments Antitumour Immunity and Boosts Anti‐PD‐1 Efficacy in Colorectal Cancer,” Gut 72, no. 8 (2023): 1497–1509. 36717220 10.1136/gutjnl-2022-328845 PMC10359538

[44] W. Liang , H. Liu , Z. Zeng , et al., “KRT17 Promotes T‐Lymphocyte Infiltration Through the YTHDF2–CXCL10 Axis in Colorectal Cancer,” Cancer Immunology Research 11, no. 7 (2023): 875–894. 37129929 10.1158/2326-6066.CIR-22-0814 PMC10320689

[45] Y. Sato , Y. Fu , H. Liu , M. Y. Lee , and M. H. Shaw , “Tumor‐Immune Profiling of CT‐26 and Colon 26 Syngeneic Mouse Models Reveals Mechanism of Anti‐PD‐1 Response,” BMC Cancer 21, no. 1 (2021): 1222. 34774008 10.1186/s12885-021-08974-3 PMC8590766

[46] Y. Jin , X. An , B. Mao , et al., “Different Syngeneic Tumors Show Distinctive Intrinsic Tumor‐Immunity and Mechanisms of Actions (MOA) of Anti‐PD‐1 Treatment,” Scientific Reports 12, no. 1 (2022): 3278. 35228603 10.1038/s41598-022-07153-z PMC8885837

