# Supplementary figures and images for: RTP4 Suppresses Colorectal Cancer Progression via MHC‐I‐Mediated CD8+ T Cell Infiltration and Enhances Immunotherapy Response
Source: J Cell Mol Med. 2025 Oct 22;29(20):e70915. doi: 10.1111/jcmm.70915 (PMC12544708; doi:10.1111/jcmm.70915)

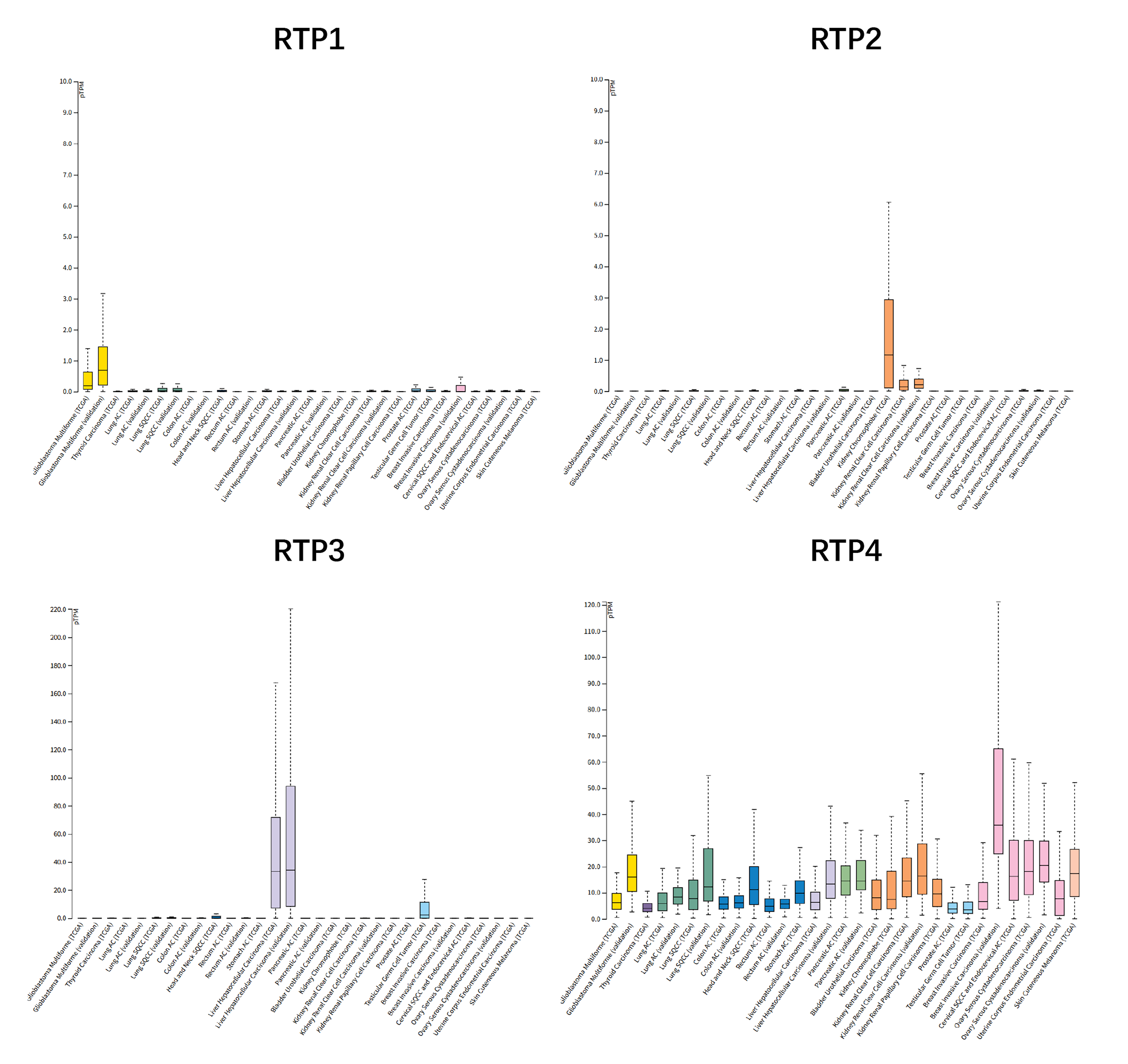

Supplement: Supplementary file 1 — Figure S1: The expression levels of RTP1, RTP2, RTP3 and RTP4 in cancer tissues across multiple tumour types. [file JCMM-29-e70915-s005.png]

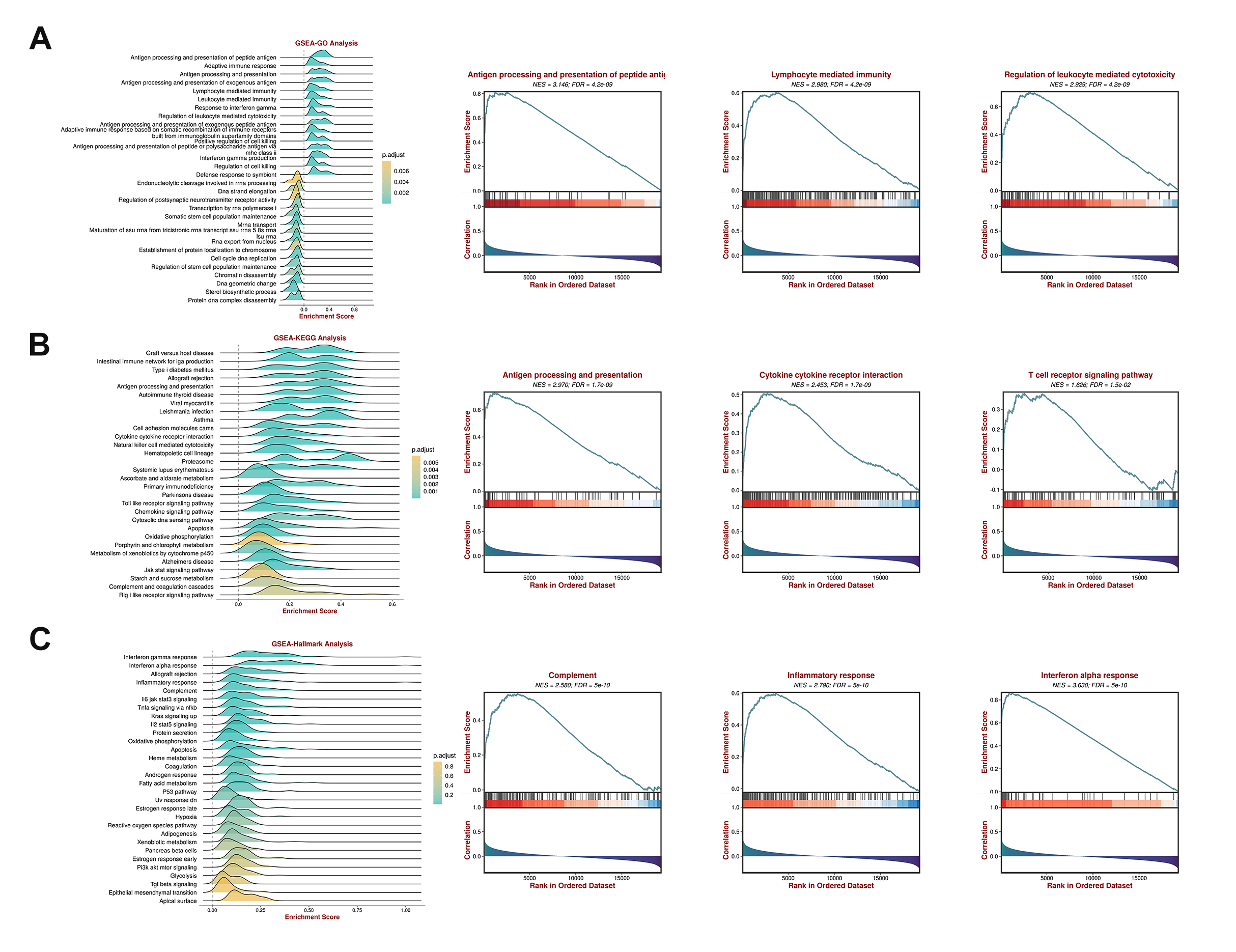

Supplement: Supplementary file 2 — Figure S2: GSEA analysis of RTP4 in CRC. (A) GSEA‐GO analysis of RTP4. (B) GSEA‐KEGG analysis of RTP4. (C) GSEA‐Hallmark analysis of RTP4. [file JCMM-29-e70915-s001.tif]

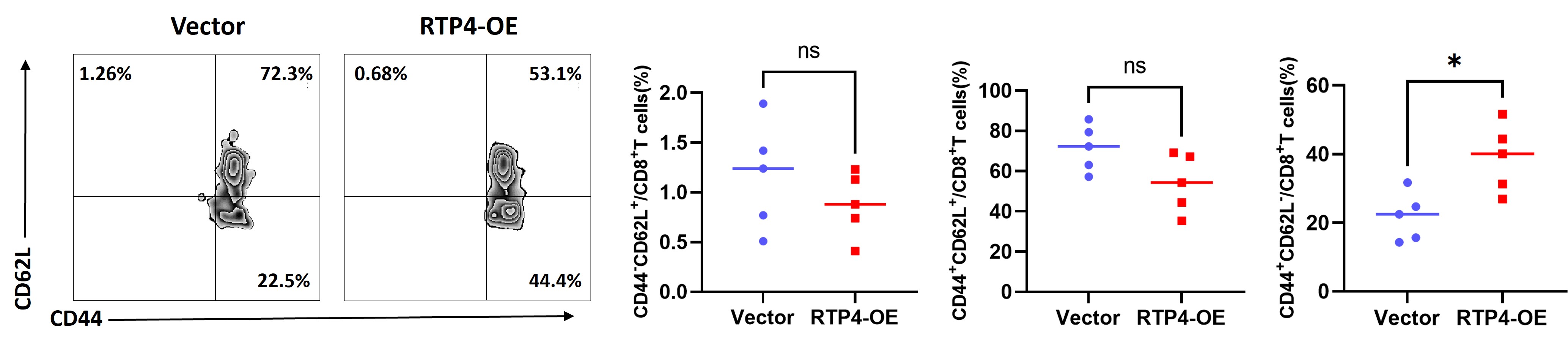

Supplement: Supplementary file 3 — Figure S3: Flow cytometry was performed to analyse the fractions of naïve (CD44−CD62L+), central memory (CD44+CD62L+) and effector (CD44+CD62L−) CD8+ T cells in MC38 tumours. *p < 0.05, ns, no significance, by two‐tailed unpaired Student's t‐test. [file JCMM-29-e70915-s002.jpg]

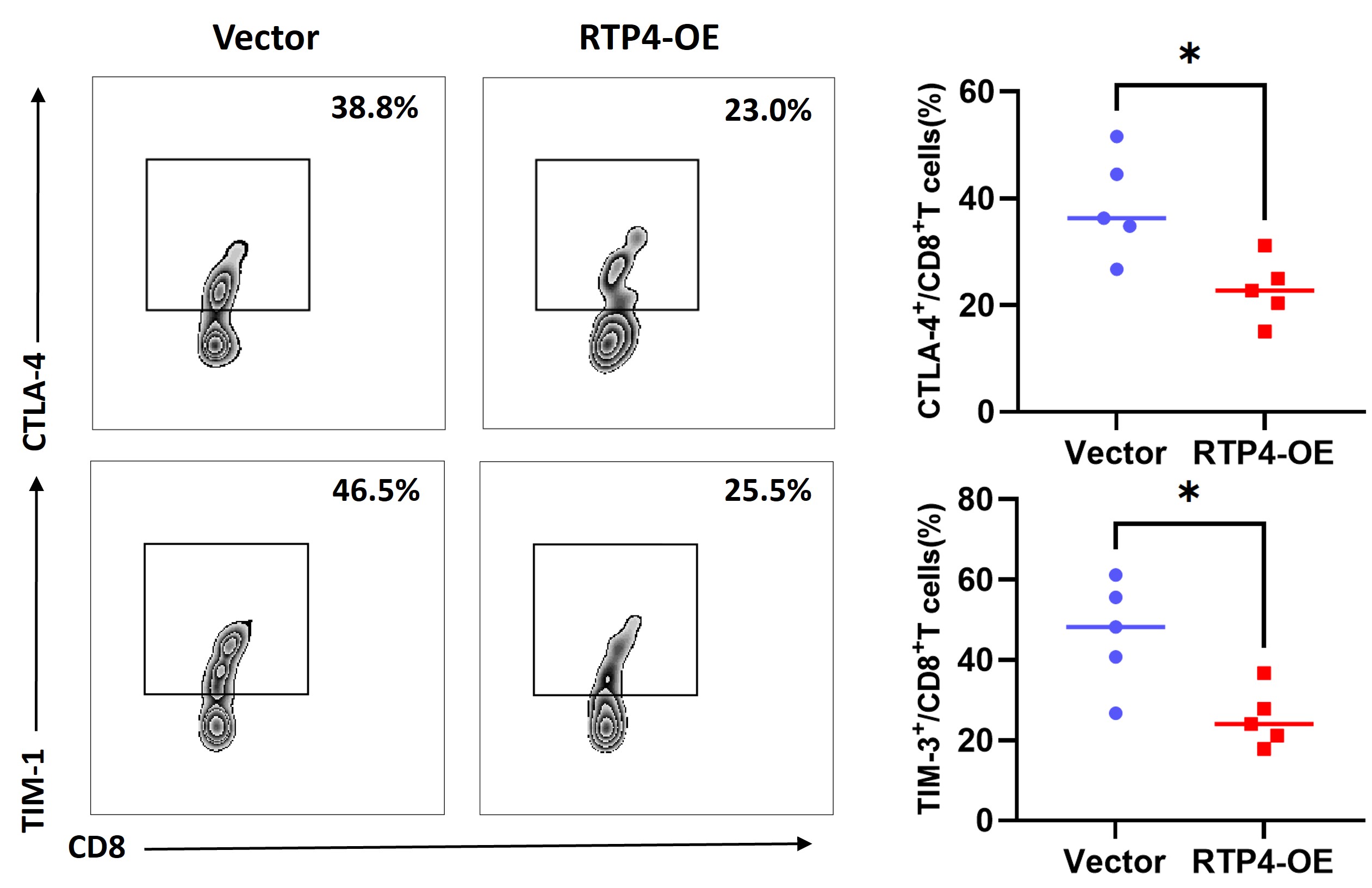

Supplement: Supplementary file 4 — Figure S4: Flow cytometry was performed to analyse the fractions of TIM‐3+ or CTLA‐4+ CD8+ T cells in MC38 tumours. *p < 0.05, ns, no significance, by two‐tailed unpaired Student's t‐test. [file JCMM-29-e70915-s004.jpg]
