# Supplementary material for: RTP4 Suppresses Colorectal Cancer Progression via MHC‐I‐Mediated CD8+ T Cell Infiltration and Enhances Immunotherapy Response
Source: J Cell Mol Med. 2025 Oct 22;29(20):e70915. doi: 10.1111/jcmm.70915 (PMC12544708; doi:10.1111/jcmm.70915)
Supplement: Supplementary file 5 — Table S1: Antibodies used in this study. [file JCMM-29-e70915-s003.docx]

| Antibody | Catalogue | Company |
| --- | --- | --- |
| Anti-DYKDDDDK (FLAG) | 20543-1-AP | Proteintech |
| Anti-GAPDH | 60004-1-Ig | Proteintech |
| anti-mouse CD16/32 | 101320 | Biolegend |
| anti-mouse CD45 | 103116 | Biolegend |
| anti-mouse CD8a | 100712 | Biolegend |
| anti-mouse CD44 | 100712 | Biolegend |
| anti-mouse CD62L | 104405 | Biolegend |
| anti-mouse PD-1 | 135214 | Biolegend |
| anti-mouse CD152 (CTLA-4) | 106305 | Biolegend |
| anti-mouse CD366 (Tim-3) | 134003 | Biolegend |
| anti-mouse IFN-γ | 505806 | Biolegend |
| anti-mouse GZMB | 372206 | Biolegend |
| anti-mouse H-2Kd | 116620 | Biolegend |
| anti-mouse H-2Kb | 116518 | Biolegend |

**Table S1:** Antibodies used in this study.
